# Glycation affects fibril formation of Aβ peptides

**DOI:** 10.1074/jbc.RA118.002275

**Published:** 2018-06-29

**Authors:** Alessandro Emendato, Giulia Milordini, Elsa Zacco, Alessandro Sicorello, Fabrizio Dal Piaz, Remo Guerrini, Richard Thorogate, Delia Picone, Annalisa Pastore

**Affiliations:** From the ‡ Department of Chemical Sciences, University of Naples Federico II, via Cintia, Napoli 80126, Italy,; § King's College London and UK Dementia Research Institute at King's College London, Denmark Hill Campus, London SE5 9RT, United Kingdom,; ¶ Universita' di Salerno, 84084 Fisciano, Italy,; the ‖ Department of Chemical and Pharmaceutical Sciences, University of Ferrara, 44121 Ferrara, Italy,; the ** London Centre for Nanotechnology, University College London, London WC1H 0AH, United Kingdom, and; the ‡‡ Department of Molecular Medicine, University of Pavia, 27100 Pavia, Italy

**Keywords:** Alzheimer disease, glycobiology, structural biology, carbohydrate, aggregation, carbohydrates, glycation, protein aggregation, fibril formation, neurodegeneration, beta-amyloid, glycosylation, amyloid plaques

## Abstract

Increasing evidence shows that β-amyloid (Aβ) peptides, which are associated with Alzheimer disease (AD), are heavily glycated in patients, suggesting a role of this irreversible nonenzymatic post-translational modification in pathology. Previous reports have shown that glycation increases the toxicity of the Aβ peptides, although little is known about the mechanism. Here, we used the natural metabolic by-product methylglyoxal as a glycating agent and exploited various spectroscopic methods and atomic force microscopy to study how glycation affects the structures of the Aβ40 and Aβ42 peptides, the aggregation pathway, and the morphologies of the resulting aggregates. We found that glycation significantly slows down but does not prevent β-conversion to mature fibers. We propose that the previously reported higher toxicity of the glycated Aβ peptides could be explained by a longer persistence in an oligomeric form, usually believed to be the toxic species.

## Introduction

The increase in life expectancy observed over the last century has led to the emergence of a new set of pathologies that constitute new challenges to scientists and clinicians. Among these, Alzheimer disease (AD)^4^ (1) has become a disorder with an increasing impact on society, because the number of diagnosed patients has more than doubled over the past 20 years and is expected to reach a prevalence worldwide of 75 million in 2030 and 131.5 million in 2050 (https://www.alz.co.uk/research/world-report-2015).^5^ Much of the increase is expected in developing countries. The fastest growth in the elderly population is taking place in China, India, and their south Asian and western Pacific neighbors. AD is the only top 10 cause of death that cannot eventually be prevented, cured, or even slowed down. Between 2000 and 2014, deaths from AD as recorded on death certificates increased 89%, whereas deaths from the number one cause of death (heart disease) decreased by 14%. Among people aged 70 or older, 61% of those with AD are expected to die before the age of 80 as compared with 30% of people without AD (2, 3).

The histological hallmark of the disease is the presence of proteinaceous aggregates, which form deposits called amyloid plaques and neurofibrillary tangles (4, 5). The main components of the amyloid deposits that accumulate extracellularly are the β-amyloid (Aβ) peptides, which are generated by a double proteolytic event by the amyloid precursor protein (APP) (6). Genetic and biochemical evidence suggests that Aβ peptides play a leading role in the etiology and progression of AD according to the amyloid cascade hypothesis (7, 8).

A key feature observed in AD patients is the presence of nonenzymatic protein glycation, because it affects long-living proteins throughout the body (9). Protein glycation, which differs from the enzyme-assisted glycosylation, starts with the condensation of a protein amino group with glucose to yield a Schiff base, which undergoes a rearrangement to form so-called Amadori reaction compounds (10). Subsequently, these species evolve by decomposition, fragmentation, and condensation and yield a widely heterogeneous set of advanced glycation end products (AGEs). Glucose and other products lead to auto-oxidation reactions, which are responsible for free radicals and highly reactive carbonyl compound production. These compounds can react, in a protein, with specific groups and contribute to post-translational modifications (10–12). The plaques in the AD brains are co-localized with the advanced AGEs, and plaque-enriched fractions contain ∼3-fold higher AGE products than preparations of the age-matched controls (13–15). A role of blood sugars would also explain the link observed between the apparently unrelated diabetes and AD; diabetic patients have a 2–5-fold higher tendency to develop AD compared with nondiabetic individuals (16, 17). Although it is clear that AGE levels increase with age, their increase in AD patients is much higher than in the average population (∼72%) (14, 18, 19).

Despite this evidence, the molecular mechanism through which glycation might influence the tendency to develop AD remains enigmatic. Glycation seems to affect the aggregation properties of polypeptides unevenly; some proteins are stimulated to aggregate by glycation, whereas glycation slows down the process for other proteins (for an exhaustive review, see Iannuzzi *et al.* (20)). Different glycation agents can also affect proteins in different ways.

Preliminary studies on the effects of glycation on Aβ have confirmed a complex scenario also for this protein. Various researchers have suggested that glycation increases the speed and the aggregate size of Aβ self-assembly (11, 12, 21, 22). Glycation by glucose or fructose has been shown to promote*in vitro* Aβ aggregation, probably because of cross-linking through AGE formation (12, 14). However, pretreatment of Aβ42 with glucose, fructose, and the glycosaminoglycan chondroitin sulfate B was shown to inhibit Aβ42-induced apoptosis and reduce neurotoxicity in neuroblastoma cells (23). The fibrillary aggregates formed upon glycation were also not cytotoxic, suggesting that glycation of Aβ could reduce its toxicity rather than increasing it.

**Scheme 1.**
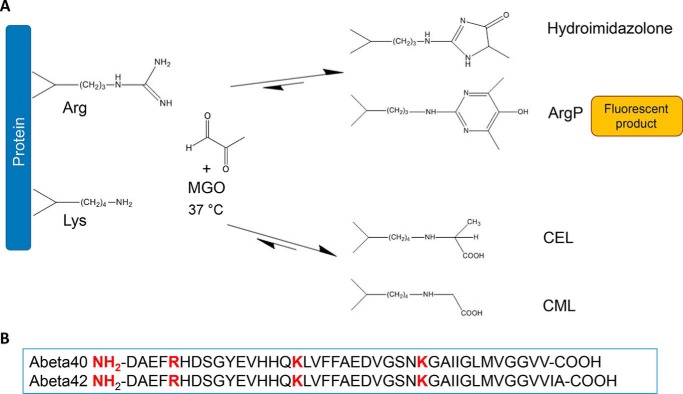
*A*, scheme of the formation of MGO glycated peptides. Glycation by MGO can occur on the protein N terminus and arginine and lysine side chains producing advanced glycation end products (AGEs), such as hydroimidazolone, argpyriminide (*ArgP*), *N*^ϵ^-carboxyethyllysine (*CEL*), *N*^ϵ^-carboxymethyllysine (*CML*), and others. ArgP emits a fluorescent signal at 405 nm when excited at 320 nm. *B*, primary sequences of Aβ peptides reporting, *in red*, the residues potentially subject to glycation.

Here, we have reconsidered the problem and addressed the effect of glycation on the two main Aβ peptides (Aβ40 and Aβ42) using an *in vitro* approach based on complementary biophysical techniques. We selected as glycation agent methylglyoxal (MGO), a by-product of the interconversion between glyceraldehyde 3-phosphate and dihydroxyacetone phosphate. MGO is a dicarbonyl metabolite that forms through multiple catabolic processes (24–28), found to be involved in AD progression. MGO glycates arginines and lysines (29), producing argpyrimidine and *N*^ϵ^-(carboxyethyl)lysine (*CEL*) and the imidazolium cross-link, methylglyoxal-lysine dimer (30) (Scheme 1). We demonstrated that, as opposed to the process observed with glucose and fructose, MGO significantly slows down formation of the mature fibers while still allowing protein aggregation and without drastically changing the morphology of the aggregates (13).

## Results

### Condition optimization

We first explored by systematically screening the effects of buffer, pH, and ionic strength on the kinetics of glycation and self-assembly to find conditions in which aggregation would be sufficiently slow to follow the glycation process using peptides synthesized in house and previously extensively characterized (31). Optimal conditions for the reaction were found using 200 mm phosphate buffer at pH 7.4, to have a polar environment in which the Aβ peptides are known to quickly aggregate and adopt a β-conformation (32). The peptides were always pretreated under strong acidic conditions (TFA) for 1 h and freeze-dried to remove the volatile acid. This protocol allows dissolving possible aggregates and thus better reproducibility between experiments. We found that optimal peptide concentrations for following kinetics within reasonable time were 100 μm. After pretreatment, the peptides were dissolved in phosphate buffer and divided into two identical aliquots, which were incubated at 37 °C in the presence and in the absence of MGO (as a control). After optimization, we found that the addition of a 100-fold excess of MGO (10 mm final concentration) was necessary to obtain efficient glycation. The vast excess of MGO may not be physiologic but was used to speed up an intrinsically slow reaction (33) and to be able to observe effects in a reasonable time span.

### Glycation of Aβ40 and Aβ42 occurs with different kinetics

**Figure 1.**
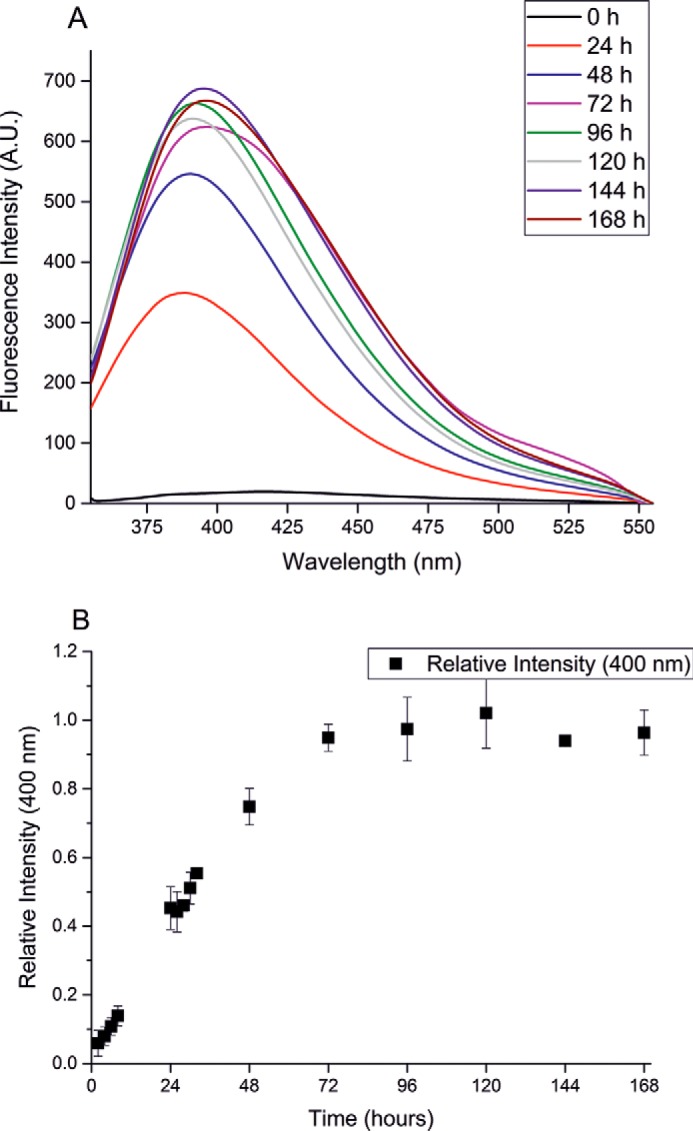
**Following glycation of Aβ40 by fluorescence spectroscopy.** *A*, fluorescence emission spectra of Aβ40G. *B*, kinetics of glycation obtaining by plotting the fluorescence intensities at the emission maximum as a function of time. The data shown are the average of three independent experiments and were normalized to the maximum of emission. When not visible, the error bar is within the *symbol. A.U.*, arbitrary units.

Aβ peptides contain an arginine in the fifth N-terminal position, which has been observed to be glycated in Aβ40 and in its fragments (34). Among the possible products, argpyrimidine is the only AGE product that fluoresces at this wavelength (35). The other potential products, such as modifications of Lys-16, do not have appreciable fluorescence emission. Glycation could thus be followed by monitoring formation of the fluorescent argpyriminide product resulting from the glycation reaction, measuring the emission spectra in the range 370–550 nm, upon excitation at 340 nm, following the procedure described by Praveen *et al.* (36). An intense fluorescence signal with the maximum centered at 400 nm was observed for the glycated Aβ40 peptide (Aβ40G) (Fig. 1*A*). The fluorescence intensity, and therefore the argpyrimidine concentration, eventually reached a plateau (Fig. 1*B*). As a reference, nonglycated Aβ40 (Aβ40NG) does not have considerable fluorescence under the same conditions, and MGO has an intrinsic fluorescence signal upon excitation at a different wavelength (data not shown).

**Figure 2.**
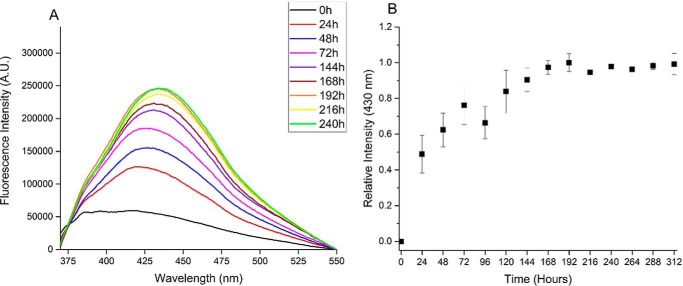
**Following glycation of Aβ42 by fluorescence spectroscopy.** *A*, fluorescence emission spectra of Aβ42G. *B*, kinetics of glycation obtaining by plotting the fluorescence intensities at the emission maximum as a function of time. The data are the average of three independent experiments. When not visible, the error bar is within the *symbol*.

The same experimental procedure was adopted to follow glycation on the longer and more aggregation-prone amyloid peptide Aβ42. Also in this case, the sample was split in two aliquots, and 10 mm MGO was added to only one of them (samples hereafter indicated as Aβ42G and Aβ42NG for glycated and nonglycated Aβ42, respectively). AGE products formed by Aβ42 had a different behavior with respect to Aβ40. During the reaction, there was a progressive increase of the fluorescence signal upon excitation at 340 nm, but the emission maximum was red-shifted as compared with the shorter peptide and centered at 430 nm (Fig. 2*A*). This behavior could reflect a different end product or a different conformation of Aβ42. The reaction was also slower than that observed for Aβ40 and reached a plateau after approximately 1 week (Fig. 2*B*). A slower glycation reaction for Aβ42 could be expected, because the glycation sites will reasonably be less accessible because this peptide is more aggregation-prone than Aβ40.

### Aβ42 has glycation reactivity similar to that of Aβ40

**Figure 3.**
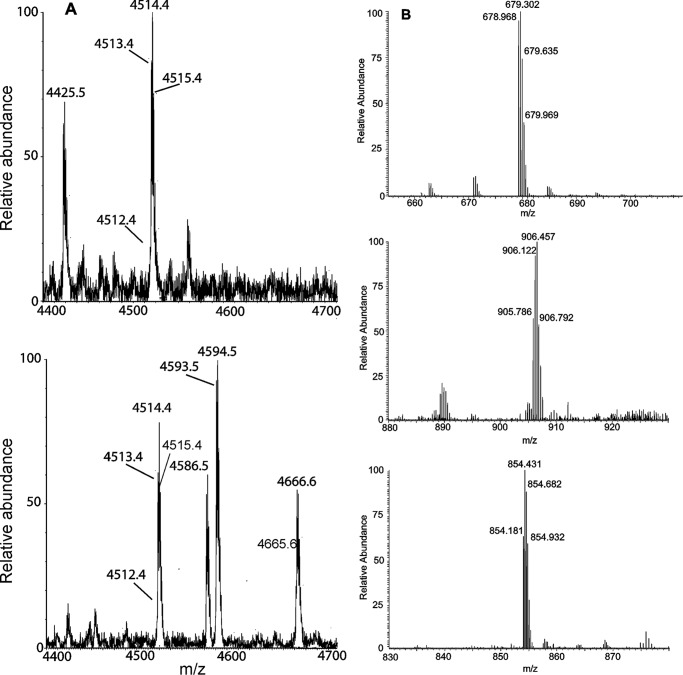
**MALDI/MS analysis to identify the glycated species formed.** *A*, spectra of Aβ42NG (*top*) and MGO Aβ42G (*bottom*). Different species observed in the spectra were identified on the basis of their molecular weight (Table 1). *B*, high-resolution ESI spectra of tryptic fragments of Aβ42G. Triply charged ion at *m*/*z* 678.968 (*top*) was generated by the fragment 1–16 carrying one argpyrimidine residue (theoretical molecular weight 2033.894). Triply charged ion at *m*/*z* 905.786 (*middle*) corresponds to the fragment 6–28 with one *N*-(carboxyethyl)-lysine (theoretical molecular weight 2714.314). The quadruply charged ion at *m*/*z* 854.181 (*bottom*) corresponds to the fragment 1–28 carrying both modifications. The difference of one mass unit between the values cited here and the figure are due to the isotopic pattern.

It has recently been described that only two of the four potential glycating groups can be glycated by MGO in Aβ40 and its shorter fragments (34). We used MALDI/MS analysis to compare these results with the behavior of Aβ42. Although chromatographic approaches are not the most suitable technique to perform an accurate quantitative evaluation of the differently modified Aβ42 species, the chemico-physical characteristics of these specific peptides make chromatographic techniques hard to use for the analysis of the undigested amyloid peptides: Aβ42 is too sticky and tends to interact strongly with the stationary phase. This results in a low yield and a low chromatographic resolution. The situation could be different for the glycated peptide, but this would, *per se*, invalidate the analysis because the quantification could enrich only one of the different species formed. On the other hand, chromatography has widely been used for semiquantitative comparisons between very similar compounds. In this light, the intensity of the signals observed in the MALDI spectra for two compounds differing only by the presence of a small group can provide reliable semiquatitative information on their relative abundance. After incubation of the peptide with MGO in phosphate buffer for 10 days, we observed three main species in addition to the unreacted peptide (Fig. 3*A*). The signal at *m*/*z* 4584.5 (mass increment of 72 Da as compared with the unmodified peptide) was presumably generated by a reaction involving the peptide N terminus or the ϵ-amine group of a lysine residue (37) (note that the difference of one mass unit between the values cited here and the figure is due to the isotopic pattern).

The ion at *m*/*z* 4592.5 (mass increment of 80 Da) indicated the formation of an argpyrimidine, as expected from the glycation of Arg-5. A minor species at *m*/*z* 4664.6 (mass increment of 152 Da) was also observed, which could correspond to the doubly modified peptide. To confirm these results and identify the modified amino acidic residues, the peptide was subjected to overnight trypsin digestion, and the fragments were analyzed by high-resolution LC-MS. Seven main species were detected (Table 1). The molecular weight of four fragments corresponded to those expected for the unmodified peptides, confirming digestion efficacy. Species with a molecular weight of 2033.903, 2714.259, and 3412.644 (Fig. 3*B*) were observed, corresponding to residues 1–16 with an argpyrimidine (theoretical weight 2033.894), fragment 6–28 with an *N*-(carboxyethyl)-lysine (theoretical weight 2714.314), and fragment 1–28 with both modifications (theoretical weight 3412.634). The identification of the glycated Lys was achieved taking into account that *N*-(carboxyethyl)-lysine cannot be recognized by trypsin (Table 2). Thus, even if peptides 6–28 and 1–28 contain two potentially reactive lysines (*i.e.* at positions 16 and 28), only Lys-16 could be glycated, as the peptide bond following Lys-28 was regularly hydrolyzed by trypsin. These results demonstrated that in full-length Aβ42, only Arg-5 and Lys-16 can be modified out of the four potentially reactive groups (N terminus, Arg-5, Lys-16, and Lys-28) and confirmed that no other fluorescence products were formed during the reaction.

**Table 1:** **List of the species identified for Aβ42 by MALDI/MS analyses** The difference of 1 mass unit between the values cited here and the figure is due to the isotopic pattern.

Ion (*m/z*)	Peptide	Experimental molecular weight	Theoretical molecular weight
4423.5	*1–41**^a^*		
4512.4	1–42	4511.4	4511.3
4584.5	1–42 (*N*-(carboxyethyl)-lysine)	4583.5	4583.3
4592.5	1–42 (argpyrimidine)	4591.5	4591.4
4664.6	1–42 (argpyrimidine + *N*-(carboxyethyl)lysine)	4663.6	4663.4

*^a^* Laser-induced fragment.

**Table 2:** **High-resolution MS analysis of the digestion products obtained by trypsin-catalyzed hydrolysis of partially modified Aβ42**

Ion (*m/z*)	Peptide	Experimental molecular weight	Theoretical molecular weight
637.298	1–5	636.291	636.287
668.810	6–16	1335.604	1335.596
663.348	17–28	1324.680	1324.666
635.386	29–42	1268.756	1268.753
678.968	1–16 (argpyrimidine)	2033.903	2033.894
905.786	6–28 (*N*-(carboxyethyl)lysine)	2714.335	2714.314
854.181	1–28 (argpyrimidine + *N*-(carboxyethyl)lysine)	3412.700	3412.675

The comparable intensity of the ions at *m*/*z* 4574.5 and 4592.5 in the MALDI spectrum also suggested that these two sites have a similar reactivity. The presence of the doubly modified peptide (observed both in the MALDI and in LC-MS analysis) indicated that glycation of one of the two sites does not significantly affect the reactivity of the other. The additional peak observed in the nonglycated Aβ42 (*m*/*z* 4423.4) was tentatively assigned to the fragment 1–41, produced during the MS analysis by the laser-induced elimination of the C-terminal alanine residue.

### Glycation affects fiber formation

**Figure 4.**
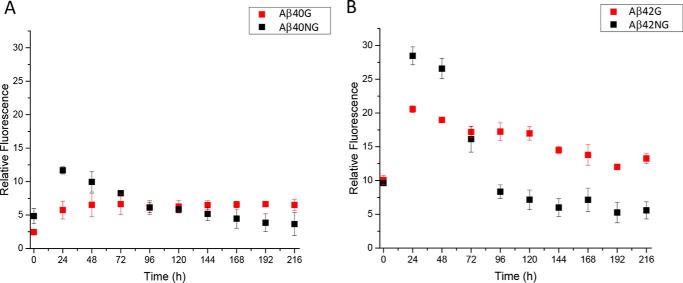
**Kinetics of fiber formation measured by the variation of the fluorescence signal of ThT.** Data for Aβ40 and Aβ42 are shown in *A* and *B*, respectively, and are the average of three independent experiments. They were expressed as -fold changes in fluorescence intensity over the background ThT signal. The progressive signal reduction likely reflects precipitation of the aggregated species.

We then adopted the widely used thioflavin T (ThT) assay to follow how glycation modifies/alters the kinetics of fiber formation (38, 39). The assay measures changes of the ThT fluorescence intensity upon binding to amyloid fibrils. It is not considered strictly quantitative (40) but nevertheless gives a reliable qualitative indication of the formation of amyloid species. We followed the fluorescence enhancement over time at 20 °C in 20 mm phosphate buffer (pH 6.8) in the presence and in the absence of MGO by fluorescence spectroscopy. In the absence of MGO, the ThT signal reached a maximum after 1 day (Fig. 4). Subsequently, the signal decayed, suggesting the formation of mature amyloid fibers, which precipitate a decrease of the signal. Overall, the fluorescence gain of Aβ42NG is larger than that of Aβ40NG. The glycated peptides have a different behavior. The ThT signals reached less intense maxima as compared with the nonglycated peptides. Decrease in time is observed for Aβ42G but is much less pronounced than for the nonglycated peptide. Noticeably, the intensity of the ThT fluorescence signal for the two peptides is very different. These results corroborated the hypothesis that glycation affects the Aβ42 aggregation pathway not by abolishing the process completely but by slowing down formation of mature aggregates.

### Sample history affects the fiber formation kinetics but does not alter protection of the glycated peptides

It is known that different batches of Aβ can behave differently due to different degrees of seeding. To verify whether our results depend on the history of the sample, we carried out in parallel the same experiments using commercial peptides. Pretreatment with TFA was carried out using the same protocol applied to the in-house synthesized peptides. We then assessed the kinetics of glycation and fiber formation using a plate reader, which allows concomitant recording of the fluorescence at two specific wavelengths and thus permits us to follow the two processes at the same time on the same sample. Under this scheme, the possibility that the data could be biased by the intrinsic fluorescence of the glycated peptides can be ruled out because the fluorescence wavelengths used for ArgP (405–420 nm) and ThT detection (485 nm) and for excitation are all very different.

**Figure 5.**
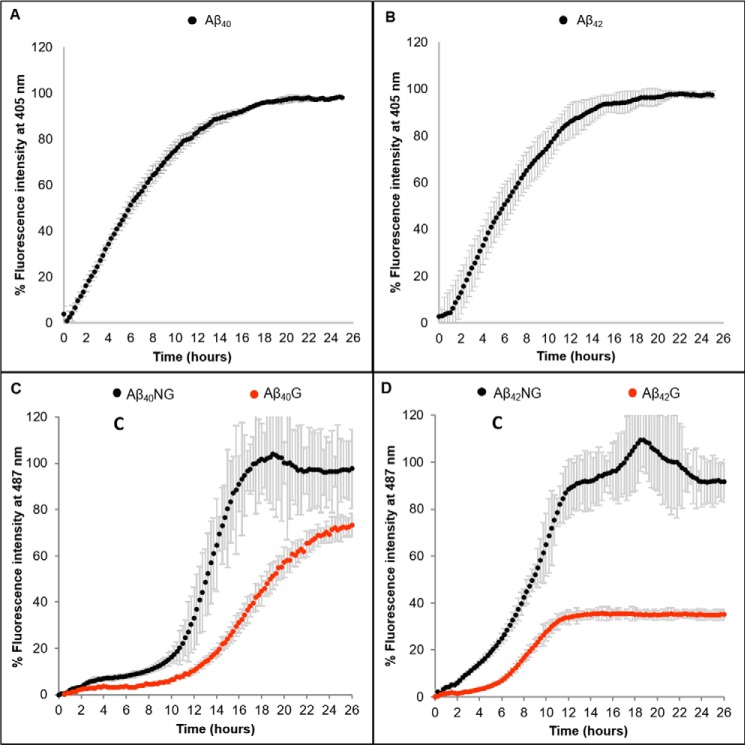
**ThT assay followed on a plate reader, which allows simultaneous recording at two wavelengths.** *A* and *B*, kinetics of glycation of Aβ with a peptide/MGO ratio of 1:100 for Aβ40 and Aβ42. The kinetics are represented proportionally to the variation of the emission fluorescence relative to formation of AGEs as a function of time. The fluorescence values were averages of three readings, normalized to the maximum value. *C* and *D*, kinetics of fiber formation of Aβ by itself (*black circles*) and with a peptide/MGO ratio of 1:100 (*red circles*) for Aβ40 and Aβ42. The kinetics are represented proportionally to the variation of the emission fluorescence of ThT as a function of time. The fluorescence values are expressed as percentages and are shown as an average of the three readings.

We observed saturation of the ThT signal at the concentrations previously used (100 μm) and had to go down to 10 μm to have reliable and reproducible measurements. This behavior remained consistent over three independently purchased batches from the same company. Both concentrations have been reported in the literature, and the differences seem to depend on the sample history (41–44). The kinetics obtained from the nonglycated peptides reached plateaus sooner and had a shorter lag phase (Fig. 5). The plateaus of Aβ42NG and Aβ40NG are also more intense than those of the glycated peptides. Despite the quantitative differences between the plate reader measurements and those on individual samples, the overall picture remained fully consistent; glycation significantly slows down fiber formation independently from the source of peptides. As expected, the effects were more marked on Aβ42 than on Aβ40. As compared with the previous measurements, we did not observe precipitation over the experiment time span, likely because of the lower concentrations used for the commercial peptides.

### Glycation slows down conversion to β-rich conformations

**Figure 6.**
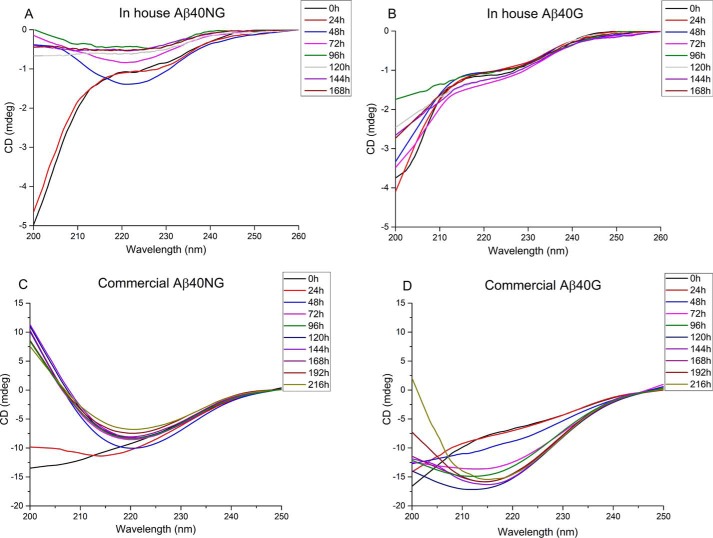
**Comparison of the CD spectra of glycated and nonglycated Aβ40 from in-house and commercial sources.** *A*, in-house–produced Aβ40NG. *B*, in-house–produced Aβ40G. *C*, commercial Aβ40NG. *D*, commercial Aβ40G. The spectra were recorded while the glycation reaction was ongoing.

The effect of glycation on the secondary structure of the peptides from both sources was then monitored by CD spectroscopy, a technique that provides information on structural conversions. We observed slightly different results. The in-house synthesized Aβ40NG peptide (at a 100 μm concentration) underwent a transition from a mainly disordered conformation to a β-rich structure, followed by an overall decrease of the signal intensity over the experiment time frame, which indicated peptide aggregation and precipitation (Fig. 6*A*). The corresponding Aβ40G showed instead a more pronounced random coil spectrum over the whole time frame, indicating that Aβ40G retained a prevalently disordered structure well after the 3 days necessary for the glycation reaction to reach a plateau (Fig. 6*B*). The signal slightly dropped in time, but the peptide did not undergo a complete transition to a β-structure.

A different but compatible scenario was observed for the commercially produced Aβ40 peptide; at 100 μm concentration, Aβ40NG peptide underwent a transition from a mainly disordered conformation to a β-rich structure after ∼1.5 days (Fig. 6*C*). The corresponding Aβ40G retained instead a random coil spectrum for longer but eventually underwent a conformational transition (Fig. 6*D*). The spectrum minimum at 215 nm remained comparatively deeper than that of the nonglycated form. Thus, independently of the source, glycation slows down transition to a β-structure.

**Figure 7.**
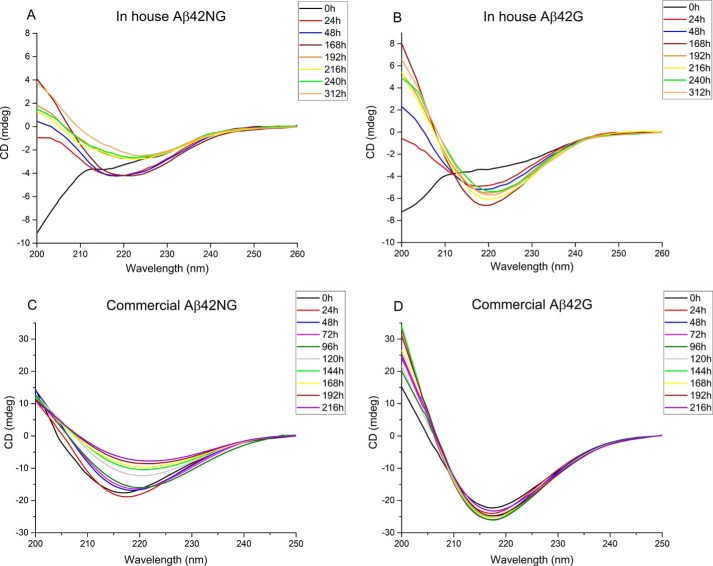
**Comparison of the CD spectra of glycated and nonglycated Aβ42 from in-house and commercial sources.** *A*, in-house–produced Aβ42NG. *B*, in-house–produced Aβ42G. *C*, commercial Aβ42NG. *D*, commercial Aβ42G. The spectra were recorded while the glycation reaction was ongoing.

The spectra of Aβ42 obtained from the two sources were only marginally different. The CD signal of the in-house–produced Aβ42NG indicated a random coil conformation immediately after dissolving the peptide in the buffer solution, but, after a short lag phase, the signal became indicative of a β-sheet structure, as testified by the unique band around 220 nm indicating aggregation (Fig. 7*A*). The minimum of this band is red-shifted with respect to the canonical value of β-structures, a behavior widely reported in the literature and explained by formation of soluble β-rich oligomers (45). The signal finally dropped, probably because of peptide aggregation. When glycated, Aβ42 underwent a transition to a β-rich structure, also in this case with a red-shifted minimum. However, after this first event, the spectrum remained invariant over time, both in intensity and line shape, for the whole time frame of the experiment (Fig. 7*B*).

The spectrum of the commercial Aβ42NG peptide was instead typical of a β-rich conformation already after peptide solubilization in buffer despite the TFA treatment, indicating that the peptide retained some degree of aggregation (Fig. 7*C*). The band minimum at 219 nm shifted slightly with time and became less intense. When glycated, the minimum became markedly deeper but retained the same intensity and position at 217 nm (Fig. 7*D*). Despite the differences between the batches of peptides, these results indicate a slower β-rich transition as a result of glycation.

### Studies to assess whether the aggregate morphology is modified by glycation

AFM was used for the three-dimensional analysis of the Aβ40 and Aβ42 aggregates as compared with their glycated counterparts to visualize the morphology of the aggregates and identify potential structural differences. The experiments were independently repeated on the peptides from both sources, resulting in qualitatively similar results (hereafter, we describe results for the more aggregation-prone commercial peptides because they showed more marked differences) (Table 3).

**Table 3:**
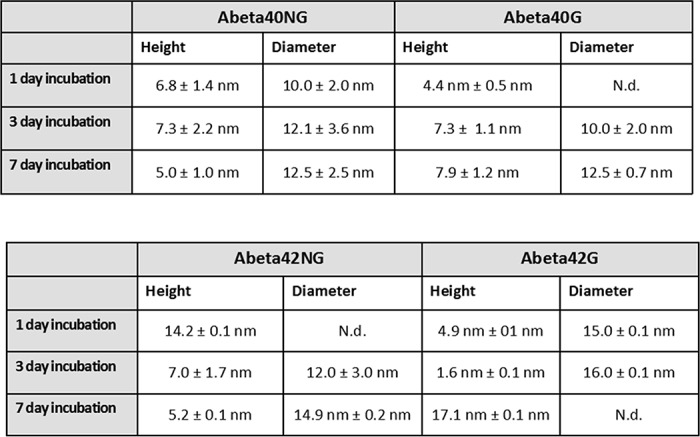
**Summary of the dimensions of the glycated and non-glycated Aβ42 and Aβ40 fibres as obtained by AFM** Heights and diameters are displayed for each time point. N.d., not determined.

**Figure 8.**
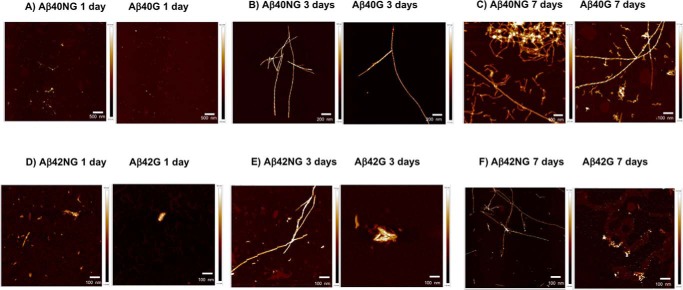
**Effects of glycation of Aβ40 (*A–C*) and Aβ42 (*D–F*) on the aggregate morphology studied by atomic force microscopy.** The micrographs show that the glycation interferes with the aggregation process by limiting the growth of the fibers and slowing down the fiber formation kinetics. Aβ40 and Aβ42 aggregates are characterized by an *equally spaced* and *twisted shape*. Images relative to Aβ40 were taken at a magnification of 5, 2, and 1 μm for day 1, 3, and 7, respectively. Images relative to Aβ42 were all taken at a magnification of 1 μm.

After a 1-day incubation at 37 °C, Aβ40NG formed large aggregates, although a few fibers were also identified. The fibers were twisted with a height of 5.4–8.2 nm and a diameter of 8–12 nm, depending on the helix pitch (Fig. 8*A*, *left panel*). The glycated counterpart seemed to form fewer multimers, which appeared to be less ordered and characterized by a height of 4.9 nm (Fig. 8*A*, *right*). At 3 days of incubation, when the reaction would be complete, Aβ40NG aggregated in long twisted fibers (2–2.5-μm length, taking into account fiber bending) equally spaced with a pitch of 70 nm (Fig. 8*B*, *left*). At the same time point, Aβ40G had the same morphology, but with a different helical pitch (35 nm) (Fig. 8*B*, *right*). After 7 days, Aβ40NG showed a higher degree of aggregation with fibers of 10–15-nm diameters and 4.0–6.0-nm heights (Fig. 8*C*, *left*). Aβ40G appeared as a mixture of shorter and thinner fibrils with longer and twisted ones (diameter of fibrils 11.8–13.2 nm) (Fig. 8*C*, *right*). Overall, the morphology of fibers formed by Aβ40 and the end point of the mature fibers did not seem to be affected by the glycation. However, we could observe fewer aggregates by AFM overall.

A similar picture resulted for Aβ42, although, overall, these samples could look less aggregated simply because of precipitation, as observed in the ThT experiments. At 1 day of incubation, Aβ42NG formed short and thick fibrils with a broad range of diameters and 14.2-nm heights (Fig. 8*D*, *left*). Aβ42G displayed only a few fibrils of smaller size (4.9-nm height and 15.0-nm diameter) (Fig. 8*D*, *right*). After 3 days at 37 °C, Aβ42NG aggregates appeared as thick and twisted amyloids with 0.8–1.6-μm lengths, 9–15-nm diameters, and 5.3-8.7-nm heights, depending on the helical pitch (Fig. 8*E*, *left*). The fibers were equally spaced with a pitch difficult to measure (65–120 nm). At the same incubation time, the glycated counterpart formed fibrils of similar diameter (16 nm) but with a significantly reduced height (1.6 nm) and length (Fig. 8*E*, *right*). After 7 days, both nonglycated and glycated Aβ42 continued to form the same type of fibers observed at day 3; they were twisted and 70–125 nm long for Aβ42NG (5.2-nm height, 14.9-nm diameter) and thick (17.1-nm height) but short for Aβ42G (Fig. 8*F*, *left* and *right*, respectively). These results thus showed that glycation with MGO interferes with the aggregation process and with fiber growth.

## Discussion

The role of the Aβ peptides in AD has been debated for years. Their accumulation in the AD brain and their role in aggregation are not currently disputed, but the exact series of events, the state that favors the process, and the precise toxic species remain unclear. The general view is that the main toxic species are the oligomeric states or soluble aggregates rather than the mature fibers (46–48). Previous studies have demonstrated that Aβ peptides are suitable substrates for glycation, a post-translational modification of increasing interest because of its association with toxic AGE species. Another aspect that makes glycation a potential important factor in AD is the observation that patients with diabetes, a disease directly linked to high sugar levels, are 5-fold more susceptible to AD than other individuals (49). This evidence suggests the urgency to enquire whether and how glycation may interfere with the structure and aggregation properties of Aβ. Here, we have addressed this important question using both Aβ40 and Aβ42, the two main species observed in AD patients (34). Being well aware of the low reproducibility of results about the highly aggregation-prone Aβ (50), we worked in parallel with synthetic peptides from two independent sources. We found systematic discrepancies in the kinetics of aggregation that could be explained by a different degree of seeding that persists despite the strong acidic treatment. However, overall, the final message remains the same: Glycation reduces the speed of aggregation.

Our results need to be evaluated by also bearing in mind that two concomitant and partially competing processes take place in parallel; glycation and aggregation of peptides as hydrophobic and aggregation-prone as Aβ co-occur. The two events can selectively be followed by detecting different fluorescence wavelengths. We found that the glycation reaction reaches a plateau faster for the shorter Aβ40 peptide than for the more aggregation-prone Aβ42. This behavior could be explained by a major availability of the glycation sites in Aβ40, although overall MS measurements on Aβ42 demonstrated that the number of glycated sites remains the same as for Aβ40.

For both peptides, the glycation reaction slows down, albeit it does not prevent completely, the transition to β-sheet structures. The aggregation-inhibitory effect is more marked for Aβ40, which, according to the CD data, remains in an unstructured state almost for the whole experiment time frame. The glycated Aβ42 peptide is able to form a β-sheet–rich structure more readily, but our CD and AFM data indicate that this species could be different from that formed by the unmodified peptide and is more soluble. ThT data of Aβ42 also indicate a less efficient conversion to the mature fibers after peptide glycation.

Our data should be put in the frame of previous results on Aβ peptides using the same or similar glycating agents. One of the first studies (11) used Aβ40, a shorter fragment (Aβ28), and glucose as the glycating agent. The authors found that after a concentration-dependent lag period of a 4-month incubation, soluble preparations of the synthetic peptides slowly form fibrillar aggregates that resemble natural amyloid and can be measured by sedimentation and ThT fluorescence. Similar seeds were prepared from the naturally occurring reaction. It was observed that AGE-modified nucleation seeds accelerated the aggregation of soluble glycated Aβ28 as compared with nonglycated seeds.

A subsequent study (21) was mainly based on a computational analysis on Aβ42 aiming to evaluate the effects of glycation on the free energy of the peptide. The authors assumed formation of two carboxymethyllysines, unfortunately unable to produce fluorescence and very different from the products we observed by MS in this study. The experimental part based on glycation by glyoxylic acid and cyanogen bromide to obtain a specific derivative could have reasonably led to other side products, which were unfortunately not discussed in the paper. These data are thus hard to compare with ours. Finally, the work by Nomomoto *et al.* (51) is concerned with biologic samples using glucose as the glycating agent, which leads to pentoxidine, a compound very different from MGO, clearly indicating how important the specific glycating agent is.

Our data correlate instead with and explain a previous study using synthetic Aβ42 glycated *in vitro* with the same glycation agent to detect toxicity on primary hippocampal neurons (13). It was observed that glycation exacerbates neurotoxicity of Aβ with up-regulation of the AGE receptor (RAGE) and activation of glycogen synthase kinase-3 (GSK-3). We propose that this behavior could be explained by considering that, according to our results, the slower process of fiber formation could have the effect of stabilizing the oligomeric state, generally believed to be the toxic species in aggregation. If true, this hypothesis would urgently solicit the identification of new effective ways to prevent sugar accumulation in the blood and reduce the risk of Aβ glycation. It will be interesting in the future to study the effect of glycation on other diabetes-related peptides, such as amylin and α-synuclein, which might unveil further important details about the relationship between these two pathologies.

## Experimental procedures

### Sample production

The Aβ peptides were either purchased from rPeptide (rPeptide catalogue nos. A1153-1 and A1163-1) or synthesized according to a previously published protocol (31). Before each experiment, they were treated with pure TFA using 100 μl/mg of peptide to dissolve pre-existent fibrillar aggregates (31). The solution was gently agitated until complete dissolution, diluted in ultrapure water and up to 10% (v/v) TFA, and freeze-dried overnight in aliquots. The aliquots were dissolved in 200 mm phosphate buffer at pH 7.4 to a final concentration of 100 or 10 μm. MGO was added in a 100-fold molar excess and incubated at 37 °C without shaking. The peptide concentrations were measured by nanodrop and a standard UV spectrometer (assuming a ϵ_280 nm_ = 1490 m^−1^ cm^−1^).

### Fluorescence spectroscopy

Fluorescence spectra of the in-house–produced peptides incubated with MGO were recorded at 20 °C on a Jasco FP 6600 and on HORIBA Fluoromax spectrofluorometers. The excitation wavelength was set to 340 nm, detecting an emission range from 370 to 550 nm and a scanning speed of 100 nm/min to follow the glycation reaction. Both excitation and emission slits were set to 5 nm. The emission wavelength was set to 430 nm in the range of 300–420 nm in excitation measurements. In ThT assays, the excitation wavelength was set to 440 nm with an emission range of 460–600 nm and a scanning speed of 100 nm/min. Both excitation and emission slits were set to 5 nm. Before each measurement, a sample aliquot was collected and diluted in 20 mm phosphate buffer at pH 6.8 to a final concentration of 10 μm, to which a 3-fold molar excess of ThT was added. All spectra were recorded at 20 °C. The data were expressed as -fold changes in fluorescence intensity over the background ThT signal according to Xue *et al.* (52). In this scheme, the fluorescence intensity of the protein sample is divided by the fluorescence intensity of the ThT-only sample.

Aggregation and glycation kinetics of the commercial peptides were followed using a FLUOstar OMEGA Lite instrument. Before each assay, the peptides were prepared as described previously. Aggregation was performed in a Greiner UV-clear 96-well plate by diluting the commercial peptide samples to 10 μm in PBS, pH 7.4, in the presence of 20 μm ThT, with or without MGO (final concentration 1 mm), to obtain peptide/MGO ratios of 1:0 or 1:100. The temperature was set at 37 °C, and the readings were performed every 15 min, setting the excitation wavelength at 440 nm and the emission wavelength at 487 nm. The plate was left quiescent between measurements and shaken for only 1 s before each reading. Glycation kinetics was followed using the same conditions as for the aggregation assays, but the reaction mixture did not contain ThT, the excitation wavelength was set to 320 nm, and the emission wavelength was set to 405 nm. In both experiments, every condition was assayed at least in triplicates. The plate reader results were expressed as -fold changes in fluorescence intensity. The overall conclusions are independent of the normalization scheme adopted.

### CD spectroscopy

Far-UV CD spectra were recorded on a Jasco J715 spectropolarimeter (Jasco, Essex, UK), equipped with a temperature control system, using a 1-mm quartz cell in the far-UV range 200–260 nm, using peptide concentrations in phosphate buffer of 10 and 100 μm for the in-house–produced and commercial peptides, respectively. Raw spectra were corrected for buffer contribution. The scanning speed was set to 20 nm/min, the average time to 4 s, and the temperature to 20 °C for all experiments. To ensure reproducibility, all experiments were repeated at least three times on at least two different batches of peptides in two different laboratories.

### Identification of the reactive glycation sites

Mass spectra of reacted and unreacted Aβ42 were acquired using a MALDI-TOF Micro (Waters) instrument operating in reflectron mode. Mass calibration was achieved using a peptide mixture derived from trypsin digestion of BSA. The peptide was digested overnight by trypsin under stirring at 37 °C, and the resulting fragments were analyzed by nano-LC-MS using a Orbitrap XL instrument (Thermo Fisher Scientific) equipped by a nano-ESI source coupled with a nano-ACQUITY capillary UPLC (Waters). Peptide separation was performed on a capillary BEH C18 column (0.075 × 100 mm, 1.7 μm; Waters) using aqueous 0.1% formic acid (A) and acetonitrile containing 0.1% formic acid (B) as mobile phases. Peptides were eluted by means of a linear gradient from 5 to 50% B in 45 min and a 300 nl/min flow rate. Mass spectra were acquired over an *m*/*z* range from 400 to 1800.

### Measurements of the fiber morphology

Atomic force microscopy was performed on samples pretreated with TFA and then dissolved in the desired volume of PBS to obtain a concentration of 100 μm. Samples were then incubated at 37 °C without shaking, and images were acquired at different time points. Height peak force error images were acquired on a Bruker Multimode 8 microscope with a Nanoscope V controller (Bruker UK Ltd., Santa Barbara, CA) operating in peak force tapping mode using ScanAsyst Air cantilevers (115-μm nominal length, 25-μm nominal width, nominal spring constants of 0.4 newtons/m, and typical resonant frequencies of 70 kHz). The ScanAsyst probes have a 2-nm nominal tip radius of curvature. Image data were acquired at peak force frequency of 4 kHz and a line rate of 3 Hz at a resolution of 512 pixels/line. Samples were diluted 1:10 and 1:100, and 100 μl of each diluted solution was added onto freshly cleaved mica and incubated at room temperature for 5 min. The excess of liquid was dried off from the mica and rinsed extensively with a gentle flux of filtered Milli-Q®-H_2_O.

### Data availability

The primary data are available from the authors upon reasonable request.

## Author contributions

A. E. and G. M. carried out the bulk of the experimental work. E. Z. and A. S. assisted and complemented their work. F. D. P. carried out mass spectrometry. R. G. synthesized the non-commercial peptides. R. T. carried out the atomic force microscopy experiments and helped in their visualization. D. P. and A. P. supervised the project, and A. P. wrote the paper with the support of all other authors.

## Footnotes

5 Please note that the JBC is not responsible for the long-term archiving and maintenance of this site or any other third party hosted site.

## Footnotes

4 The abbreviations used are:

AD: Alzheimer disease
Aβ: β-amyloid
APP: amyloid precursor protein
AGE: advanced glycation end product
MGO: methylglyoxal
ThT: thioflavin T
ESI: electrospray ionization.
